# When home becomes the office: navigating challenges and embracing possibilities in telework in Sweden during and after the COVID-19 pandemic

**DOI:** 10.3389/fpsyg.2025.1516074

**Published:** 2025-07-09

**Authors:** Paraskevi Peristera, Christine Bergljottsdotter, Constanze Leineweber

**Affiliations:** Department of Psychology, Stockholm University, Stockholm, Sweden

**Keywords:** telework, work life balance, wellbeing, health, qualitative study, hybrid work

## Abstract

**Introduction:**

The COVID-19 pandemic was a disruptive event that forced employees worldwide to quickly shift to telework. This qualitative study explored employees’ experiences of telework during and after the COVID-19 pandemic in Sweden, where a more liberal approach to restrictions and telework was taken, focusing on changes in perceptions of work, work–nonwork interplay, relationships, wellbeing, health, and work–life balance.

**Methods:**

Semi-structured interviews, which were transcribed verbatim using Amberscript, were conducted with 16 participants from the SLOSH-Corona survey, who teleworked during the COVID-19 pandemic and continued to telework to varying extent after the removal of restrictions.

**Results:**

Reflexive thematic analysis, based on Braun and Clarkes six step, identified five main themes: (1) having what it takes: the hoffice; (2) all work and no play: efficacy and loneliness; (3) faces of flexibility: freedom and balancing boundaries; (4) leadership challenges: bridging the gap between employee- and organizational needs; (5) survive or thrive? Telework and quality of life. Overall, telework was associated with high work efficacy. Additionally, increased work flexibility combined with effective management of work-nonwork boundary and strong supervisor support improved work-life balance, wellbeing, and quality of leisure time. However, work intensification was also high, as well as work-related isolation, ergonomic health problems, and sickness presence.

**Discussion:**

For future telework to be sustainable, organizations would benefit from providing employees with home-based work supplies, and in particular, implementing leadership based on trust, enhanced work-related social connection, and organizational norms supporting clear work-nonwork boundaries.

## Introduction

The COVID-19 pandemic changed working life for an extensive number of employees worldwide, as telework—also referred to as remote work or work from home—became the norm. This arrangement involves performing job duties outside traditional office settings, typically from home, using digital technologies to stay connected. These changes still affect how we work today, since telework—either entirely from home or a combination of work-from-home and on-site-work continues; at least to some extent. In Sweden, the prevalence of workers who work mostly from home increased from 3 to 4% before the pandemic to over 26% in 2021 and decreased to a level around 13–16% after the removal of restrictions (Ekberg and Beijron, 2024). This means that the proportion of employees who mainly work from home has increased by approximately 10% points since the pandemic. It is also estimated that around 1.8 million of employees in Sweden today combine work-from-home and on-site-work (Kreicbergs and Ohlin, 2024).

Teleworking has introduced both notable advantages and significant challenges. Among the most commonly reported benefits are increased flexibility and autonomy, greater control over one’s work environment, reduced commuting time, cost savings, and, for some, improved work–life balance (Chan et al., 2022; ILO, 2023). It has also been seen as environmentally beneficial due to decreased travel. In contrast, disadvantages include social and professional isolation, blurred boundaries between work and nonwork life, digital overload, technological and ergonomic difficulties, reduced career visibility, and unequal access to telework opportunities across different sectors and socioeconomic groups (D’Abundo et al., 2023; European Agency for Safety and Health at Work, 2023). These telework-driven dynamics, especially when combining work from home and on-site work, have given rise to new and complex challenges for both organizations and employees, particularly concerning work–life balance (WLB), health, and overall wellbeing.

This new working situation has led to a great deal of uncertainty among organizations since they, to an even greater extent than before, have to balance organizational goals with employees’ evolving needs and expectations (Danielsson, 2024). In this context, although research is still scarce (Chan et al., 2022; European Agency for Safety and Health at Work, 2023), organizations are being called to implemate a set of technical, organizational, and managerial transformations with as yet unknown results for employees’ job satisfaction, wellbeing, mental health at work, and work-life balance (WLB) (European Agency for Safety and Health at Work, 2023; Sonnenschein et al., 2022).

The overall concept of WLB comprises of both work-to-family conflict (WFC) and family-to-work conflict (FWC), as well as enrichment and facilitation (Greenhaus and Beutell, 1985). Conflict implies that employees experience an inter-role conflict (Frone et al., 1997) that arises when demands from the work and nonwork domains are mutually incompatible (Geurts et al., 2005; Greenhaus and Beutell, 1985) as the individual experiences insufficient time and/or energy, i.e., resource scarcity, to perform work and family roles successfully (Frone et al., 1997; Michel et al., 2011; Netemeyer et al., 1996).

Research has acknowledged that WLB is of particular importance for employees who telework since it can contribute to a healthy, stress-free (work) environment and improved wellbeing (Feeney and Stritch, 2019; Vyas, 2022). With the onset of COVID-19 and subsequent lockdowns and implementation of telework, the boundaries between work and nonwork altered with implications for both WFC and FWC. Research findings are however heterogeneous with some studies highlighting the blurring of work-nonwork boundaries, leading to increased conflicts between the work- and nonwork domains (Allen et al., 2021; Chan et al., 2022; Howe et al., 2021; Johnston et al., 2023; Reimann et al., 2022). Other studies have instead shown reduced conflicts between work and nonwork and improved WLB (Alfano et al., 2023; Elhinnawy et al., 2023; Schieman et al., 2021). Regarding telework in the post-pandemic era, where the combination of both work-from-home and on-site-work is predominant, some studies found that work arrangements with 1–2 days telework per week may have a greater positive effects on health, wellbeing and WLB than full-time telework (Hopkins and Bardoel, 2023; ILO, 2023; Teng-Calleja et al., 2024). Others suggest that combining telework and on-site-work does not always facilitate the combination of family, social and professional demands (Antunes et al., 2023; Elbaz et al., 2023; Teng-Calleja et al., 2024). Although these recent findings indicate that opportunities as well as challenges differ for full-time teleworkers and those that combine telework with on-site work, most of the existing studies have ignored the complexity of the latter (European Agency for Safety and Health at Work, 2023).

In addition to WLB, telework during the pandemic also seems to affect job characteristics and wellbeing. For instance, telework was found to be related to higher job demands, longer and more intense working hours, and increased turnover, but also improved job satisfaction (Chan et al., 2022; Kwon and Kim-Goh, 2023; Weinert and Weitzel, 2023). The evidence on health and wellbeing is mixed: some studies suggest that telework contributes to stress, emotional exhaustion, burnout, musculoskeletal problems, and overeating, while others indicate reduced stress, improved sleep quality, and fewer psychosomatic symptoms (Corrente et al., 2024; Fadel et al., 2023; George et al., 2022; Gualano et al., 2023; Hsu and Engelhardt, 2024; Perelman et al., 2024). Additionally, telework has been associated with increased risk for social isolation (D’Abundo et al., 2023) as well as overall overload, especially for employees with children (Rudolph et al., 2021).

While the effects of full-time telework during the pandemic are relatively well researched, knowledge remains limited regarding how hybrid work arrangements—those that combine work from home and on-site work— affect employees’ job characteristics, wellbeing, health and WLB in the post-pandemic comtext. Some studies have shown that teleworking a few days per week may have greater positive effects on wellbeing and quality of life than full-time telework (European Agency for Safety and Health at Work, 2023; Juchnowicz and Kinowska, 2021). Other studies have shown that full-time telework or exclusive on-site work associates with poorer mental health compared to work arrangements with both telework and on-site work (Bodner et al., 2022). Since the association between different telework arrangements and WLB, wellbeing and health may vary considerably, a call has been made for research that accounts for the complexity of different telework arrangements (European Agency for Safety and Health at Work, 2023; Hall et al., 2023; ILO, 2023). Various methodological limitations of pandemictelework research that reduce its applicability to the post-pandemic era have also been discussed. For instance, the lockdowns due to the COVID-19 pandemic and the sudden and mandatory transition to telework without prior preparation may have exacerbated the negative effects on both work and health outcomes. Therefore, mid-pandemic findings may not fully disentangle the respective effects of telework from those of the economic, social, and health contexts related to the COVID-19 crisis. Furthermore, employees that combine telework with on-site work are exposed to risks and advantages related to as well full-time telework as full-time on-site work and therefore pandemic research from countries who employed strict lockdowns and mandatory full-time telework cannot provide information that could be extrapolated to telework in the post-pandemic era (European Agency for Safety and Health at Work, 2023).

Sweden, in comparison to other countries, adopted a relatively liberal approach to restrictions and lockdowns during the pandemic (Ludvigsson, 2020; Pashakhanlou, 2022). The Swedish recommendations implied working from home when possible, self-imposed social distancing, self-monitoring for symptoms, staying home when ill, socializing outdoors and only with a small number of people, practicing good hand hygiene, and distance education at high school (in some periods) and universities. This implies that individuals in Sweden had to make their own judgments on how to act and make choices in everyday life. Evidence from Sweden, where instead of lockdowns voluntary restrictions on social distancing and teleworking were applied, could thus provide useful information for post-pandemic telework arrangements. The few existing Swedish studies on telework during the pandemic have reported increased likelihood for higher job satisfaction but also less spontaneity during digital meetings (Brogårdh et al., 2021; Espersson et al., 2023), and positive effects of increased flexibility in work- and leisure activities as well as on health and wellbeing (Lindgren et al., 2023). Still, there is a knowledge gap, not only in the Swedish context, regarding the role of telework as it is implemented in the post-pandemic era, for employees’ overall job satisfaction, wellbeing, health, and WLB (Chan et al., 2022; European Agency for Safety and Health at Work, 2023).

Therefore, the present qualitative study aimed to investigate employees’ experiences of telework during and after the COVID-19 pandemic in Sweden. Specifically, this study explores potential changes in how employees perceivetheir work, manage the interplay between work and nonwork, and experience relationships, wellbeing, health, and WLB in a hybrid work landscape.

## Theoretical framework

### Boundary theory

The present study adopted boundary theory in order to answer the aim stated in the above. Within psychology and organizational research, there has even before the pandemic been an increased interest in how boundaries between work and nonwork are constructed, demarcated, and maintained. From a boundary perspective, individuals construct boundaries, both psychologically and behaviourally, in order to organize their work and nonwork domains (Bulger et al., 2007; Clark, 2000; Matthews and Barnes-Farrell, 2010; Nippert-Eng, 1996). These boundaries can be analysed along a segmentation-integration continuum where employees have preferences for keeping various aspects of work-nonwork more or less separated from one another, i.e., segmenting, or integrating work and nonwork, i.e., the degree to which various aspects of work-nonwork are merged or blended (cognitively, behaviorally, and/or physically) (Ashforth et al., 2000; Clark, 2000; Kossek et al., 2006; Kreiner, 2006; Kreiner et al., 2009; Nippert-Eng, 1996). The degree of permeability is connected to the extent to which work and nonwork penetrate one another (Hecht and Allen, 2009), that is, the frequency with which individuals cognitively or behaviourally shift their resources (time or attention) from one domain to another by engaging in specific actions. Both boundary management strategies, that is, segmentation and integration, have been found to bring about costs and benefits. For instance, segmentation can be beneficial when it comes to fulfilling work and nonwork roles (Dumas and Sanchez-Burks, 2015) and reducing WFC (Powell and Greenhaus, 2010). In some cases, however, segmentation can lead to more WFC, since integration, although more difficult, sometimes may be necessary in order to combine work and nonwork activities (Ashforth et al., 2000). Integration has however more often been shown to be problematic as it relates for example to longer weekly work hours, poorer WLB (Mellner et al., 2014), more cross-role interruptions (Ashforth et al., 2000), more WFC (Kossek et al., 2006; Matthews et al., 2014; Mellner et al., 2021), and greater inter-role conflict (Bulger et al., 2007; Hecht and Allen, 2009). These earlier findings are further supported by a recent study (Mellner et al., 2021) showing that integration was associated with the second highest levels of WLC, even when this was the preferred strategy of the employee. In contrast, boundary congruence, that is, enacting one’s preferred strategy, in terms of enacting and preferring segmentation, was associated with the lowest levels of WLC. Employees not enacting their preferred strategy, i.e., boundary incongruence, also reported high WLC, especially those preferring segmentation but enacting integration who reported the highest levels of WLC. This means that enacted integration was problematic for WLC, irrespective of one’s preferred boundary strategy. In addition, perceived control over one’s work-nonwork boundaries moderated the relationship between boundary incongruence and WLC, especially for those with a preference for segmentation but enacting integration. Hence, although enacting one’s preferred boundary strategy may not always be possible, perceived boundary control was found to be vital for WLC. These findings are in line with the few existing studies on the role of perceived boundary control between individuals’ work-nonwork boundary management and both WLC (Kossek et al., 2006) and WLB (Kossek et al., 2012; Mellner et al., 2014).

Given the account in the above, and in context of the present study, particularly employees in telework, where a large part of work is performed from home, may experience blurring of work-nonwork boundaries, which in turn, can put increased demands on successfully navigating the borderland between the work and nonwork domains with possible implications for overall job satisfaction, wellbeing, health, and WLB.

## Methods

The study was approved by the regional ethics committee in Stockholm (DNR 2022-01927-01).

### Participants and procedure

In this study we used a qualitative study design to investigate employees’ experiences of telework during and after the COVID-19 pandemic in Sweden. The participants were chosen from the SLOSH-Corona survey, a sub-sample of the Swedish Longitudinal Occupational Survey of Health (SLOSH). SLOSH is a large cohort survey, approximately nationally representative of the Swedish working population (*N* = 51,412), including participants from all labour market branches and occupations. For a more detailed description see Magnusson Hanson et al. (2018) or www.slosh.se. To achieve a varied and representative study sample, the following sampling strategy was employed: From the 11,310 participants who provided contact information in SLOSH-2022, we selected those who had reported teleworking at least 75% of their working time in the SLOSH-Corona survey (*n* = 320). SLOSH-Corona is a web-based survey specifically designed to capture work- and health-related experiences and changes resulting from the COVID-19 pandemic. The SLOSH-Corona survey was distributed in two steps In a first step, in January/February 2021 the web-survey was sent to 3,041 SLOSH participants (response rate: 63%, *n* = 1,903). In a second step, in October/November 2021, an additional 700 individuals from the broader SLOSH cohort were invited, bringing the total number of invited participants to 3,741 (overall response rate: 82%, *n* = 1,580).

We further reduced the sample by selecting those who stated in the SLOSH 2022 survey that they worked at least 30% of full time (*n* = 201) and were 65 years old or younger (*n* = 187). Further, among these, we excluded participants who participated in other ongoing sub-studies of the SLOSH, which left us with *n* = 181 individuals in the sample. From these 181 individuals, we conducted semi-structured in-depth interviews with 16 participants until saturation was achieved, based on a selection targeted at attaining diversity regarding gender, age, family type, place of residence, industry, as well as the extent of telework after the pandemic (see Appendix).

The participants were first contacted via email. They were given information about the study and told that they would be contacted within a week by telephone to arrange an interview, provided they agreed to participate in the study. The interviews were carried out in April–May 2023, approximately 1 year after the COVID-19 pandemic recommendations on telework ended in Sweden, although many of the participants still worked from home to a high extent.

The interviews were conducted via Zoom (Version 6.1.12) (Zoom Video Communications Inc, 2016), except for three interviews that took place over the telephone. The interviews lasted 40–60 min, were tape recorded, transcribed verbatim using Amberscript, and then erased. The interviews were based on a semi-structured interview guide focusing on how participants experienced telework during and after the COVID-19 pandemic. Specifically, the research questions targeted potential changes in perceptions of their work, the interplay between work and nonwork, work relationships, and health. Validity was ensured through investigator triangulation, reflexivity and member reflections.

### Analysis

Reflexive thematic analysis (RTA) was used to analyze the interview material. The goal of RTA is to identify and analyze patterns, or themes, in a given data set (Braun and Clarke, 2006, 2012). In RTA, the researcher is considered to play an active role in knowledge production where his/her subjectivity and reflexivity are used as resources (Braun and Clarke, 2019). Regarding the understanding of the phenomenon at hand in the present study, the second author, CB carried out all interviews and conducted the initial analysis. CB’s earlier research has focused on employees’ management of work-nonwork boundaries, and the relationships with various health-related outcomes and work-life balance. Hence, the understanding of the contextual factors in the results was enhanced. Moreover, all three authors, PP, CB, and CL, in their respective professional roles as university professors and researchers, teleworked during the COVID-19 pandemic, and had different experiences of and preferences for this way of working. Finally, although CB conducted the main part of the analysis, discussion with the two other authors, PP and CL, was important throughout the analytical process in order to ensure that the influence of CB’s subjectivity was acknowledged, which allowed for a careful analysis and interpretation of data (Frost, 2016). This, in turn, ensured that theoretical assumptions constituted an active element of the analysis.

The analysis was conducted through a constructionist paradigmatic framework (Braun and Clarke, 2021), where meaning and meaningfulness are regarded main criteria in the coding process (Byrne, 2022). A predominantly inductive, or “data driven,” approach was adopted, meaning that data was open-coded and data/respondent-based meanings were emphasized. A degree of deductive, or “theory-driven,” analysis was however employed in order to ensure that the open-coding contributed to the production of themes that were theoretically meaningful to the research questions. A combination of both approaches is often the case in coding and analysis (Braun and Clarke, 2012, 2019, 2021). Latent coding was utilized, i.e., codes represent the researchers’ attempts to identify hidden meanings or underlying assumptions that may inform the semantic content of the data.

The analytical process included six steps (Braun and Clarke, 2006, 2012): (1) reading and re-reading the interview transcripts in order to get familiar with the data; (2) generating initial codes through coding of each segment of the data that was relevant to the research questions; (3) searching for themes in terms of organizing codes into broader themes that said something specific about the research questions; (4) reviewing potential themes by modifying and developing the preliminary themes identified in the previous step and re-reading the data associated with each theme and considering whether the data supported it; (5) defining and naming themes by answering questions such as what the themes are saying, if there are sub-themes, how the themes relate to each other; and finally, (6) writing up report.

## Results

Five main themes were generated from the reflexive thematic analysis. The main themes and their respective sub-themes are presented in Table 1. Each theme is presented below and exemplified with illustrative quotes with clarifications in brackets ().

**Table 1:** Themes and sub-themes as a result of the application of reflexive thematic analysis.

Main themes	Sub-themes
Having what it take: the hoffice	Ergonomic- and technological work supplies; couch surfing’ vs. separate work space
All work and no play: efficacy and loneliness	Focus vs. creativity; communication and social relations
Faces of flexibility: freedom and balancing boundaries	Time-spatial flexibility; work-life boundaries and balance
Leadership challenges: bridging the gap between employee- and organizational needs	Trust vs. control; employee preferences and performance measurement
Survive or thrive? telework and quality of life	Leisure and lifestyle; recovery and wellbeing

**Figure 1:**
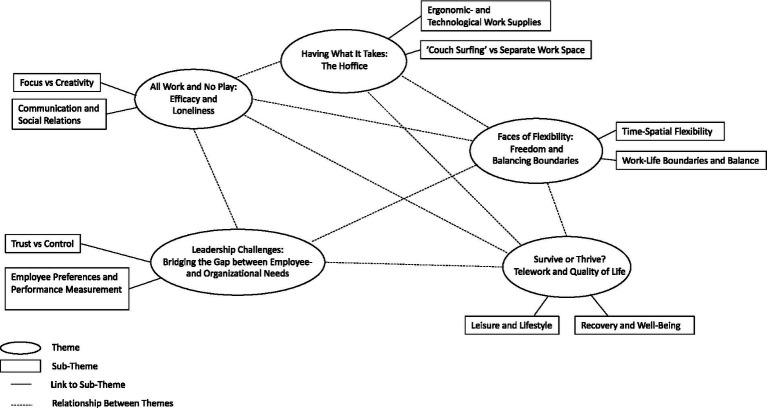
Thematic map of themes generated through reflexive thematic analysis.

In Figure 1 the thematic map illustrates the interactive and interdependent nature between the five main themes.

### Having what it takes: the hoffice

Overall, this theme noted prerequisites for teleworking and shed light on how access to, or lack of, these was experienced by the participants. This theme was comprised of two sub-themes. The first sub-theme considered issues around the (in)accessibility to ergonomic- and technological work supplies required when working from home. A second sub-theme denoted the role of having a separate work space in the home during mandatory telework.

### Ergonomic- and technological work supplies

There was a shared experience among the participants that ergonomic- as well as technological work supplies, i.e., ergonomic chairs, adjustable-height or sit-stand desks, desktop computers, lap tops, key boards, smartphones, and stable internet connection, are crucial when working from home in order to enable a sustainable physical work environment.

*“I have a good workplace at home with an adjustable-height and sit-stand desktop, and a big screen, where I can put the computer and stand at a work spot that’s equally good as when I was at work (the work place).”* (IP16)

Regarding technological equipment, specifically having a smartphone, computer/lap top, and a stable internet connection, this was deemed crucial in order to be able to perform one’s work at all when working from home. In relation to this, a vast majority of the participants underscored that having this kind of equipment is what matter for their work, whereas one is highly independent of where work is performed, whether it be at the workplace or from home.

*“I am a system developer so I want great equipment at home and extra screens and a good chair and external key boards and mice and head phones and all that. So, I actually have better equipment at home than at most offices where I am at.”* (IP14)

Access to work supplies for telework however differed among the participants. For some, the employer provided ergonomic chairs, adjustable desktops, and desktop computers, big screens and/or double screens, and in some cases also had this delivered to the employee’s home. Others had to arrange with this kind of work supplies for themselves, either using what they had at home, for instance stapling books at a table in order to get the lap top in eye level, or buying the needed supplies themselves.

*“I purchased an adjustable-height desktop online. I put a big screen, the one that I got here, on a small build-up so that it’s in eye level. I purchased a key board that you can connect to it. I have made a workplace with a proper chair too./…/My supervisor was okey with that. They (the employer) knew I had to buy myself a workplace. I didn’t get any compensation for the desktop, but they (the employer) paid for the big screen and the key board.”* (IP12)

### Couch surfing’ vs. separate work space

At the beginning of mandatory telework during the COVID-19 pandemic, the participants expressed that they had the expectation that this was just a temporary arrangement due to an extraordinary situation. However, when having worked from home during a longer period of time, a growing realization of the necessity for a separate workspace in the home emerged. This was largely due to practical aspects related to the sub-theme described in the above, i.e., ergonomic- and technological work supplies, where the need for proper equipment required for a sustainable physical work environment, but also having work things collected at one place and not spread all over the home, increased over time. For co-habiting participants, the need for a separate workspace was furthermore related to not disturbing each other if several family members worked from home at the same time. As such, over the course of mandatory telework during the COVID-19 pandemic, many of the participants created separate workspaces in the home.

*“At the beginning (of mandatory telework), I sat by the kitchen table with my computer and thought “I can sit here. That’s no problem.” But when you have done that for some weeks you realize that it doesn’t work./…/And both of us (participant’s partner) worked from home during this time period and we realized that we were climbing on top of each other.* (IP13)

Depending on socioeconomic factors, such as household size, family situation, and income, there was however variation among the participants regarding possibilities for creating a separate workspace in the home. For those living alone and/or having a larger living space, a separate workspace was more or less a given even before the COVID-19 pandemic. This was especially the case for participants in the upper middle-age, dual-earner couples, couples with grown-up children, and high-income earners.

*“Now I sit in what has become my home-office and it’s a guest room where we have a bed for guests and things like that, and a small desktop and some book shelves. So that’s what I annexed as a workspace.”* (IP3)

A different situation was identified for participants who were low-income earners, those living in small apartments, having several children, as well as for single parents. For them having a separate workspace in the home was not an option. As such, they had to perform their work where ever in the home it was possible irrespective of whether that space was ergonomically adapted or not, as well as having to change workspaces throughout the working day depending on other, competing activities in those spaces, such as when cooking in the kitchen or moving out of the children’s room after the school day was finished.

*“I mostly sat in my son’s room because he had two big screens and a gaming chair…/But every other week, when he was living with me, and he would come home at around 2–3 pm, he kicked me out of his room.”* (IP7)

Taken together, this theme highlighted variations in the access to ergonomic- and technological work supplies. This was explained by work-related factors, such as the extent to which employers provided ergonomic and technological work supplies, and by socioeconomic factors of relevance for having the means to create a separate workspace in the home, such as income, family status, and living space. In this way, there was not an even distribution of the prerequisites needed for well-functioning telework, and the participants moreover had little to no influence over the factors determining the (in)accessibility to these prerequisites.

### All work and no play: efficacy and loneliness

This theme revolved around issues regarding potential changes in work during mandatory telework, and whether certain work tasks can be considered more or less suited for telework. Two sub-themes were identified. The first sub-theme concerned an overall experience of getting more work done, and to a higher quality, during the working day when teleworking as compared to working at the regular workplace. A second sub-theme denoted a sense of missing out on work-related communication- and information as well as a lack of social exchange with colleagues during telework.

Overall, a majority of the respondents expressed that their work content- and tasks had not changed during mandatory telework during the COVID-19 pandemic, as their work per se could be performed more or less irrespective of location.

*“There’s no difference as I work with people from all over the world. They have no idea of where I am at and nobody’s asking either.” (IP1)*

What did change during mandatory telework during the COVIDovid-19 pandemic, however, was meeting formats which overnight went from physical to online meetings, using applications such as Zoom or Teams. In this way, the time it usually took to get from one meeting to another was reduced to a minimum. This, in turn, led to that it was common to schedule meetings back-to-back, resulting in the working day being filled with meetings. This was described as leading to increased efficacy in work, but also to fewer breaks and less time spent on socializing with colleagues.

*“You barely have the time to stand up before the next meeting starts.”* (IP2)

### Focus vs. creativity

The ability to focus better on one’s work during telework as compared to being at the regular workplace was explained by the fact that when working from home one is not disturbed by surrounding factors, for instance colleagues coming by one’s workspace/office, asking about something work-related or just wanting to socialize for a moment.

*“I became more efficient when I got to write my reports in peace and quiet, when no one came and knocked on the door (like at the workplace) and wanted to ask something, it was a huge difference for me.”* (IP11)

This experience was especially pronounced by respondents who worked in an open office space at the regular workplace. In this kind of office design, various kinds of factors were described as interrupting one’s work focus, such as visual motion and sounds as there were always people moving around.

*“I have difficulty not hearing what other people talk about/…/then I get disturbed and need ear phones./…/At home, I didn’t need to bother about that, because the environment was super calm.”* (IP8)

Moreover, there was the perception that certain work tasks were more suited to telework than others. In particular, mental tasks requiring concentration, for instance conducting analyses and writing different kinds of reports, were considered optimal for telework.

*“Everything that is individual work is almost always better to do from home/…/…administration, accounting, working in excel-files, things which requires peace and quiet.”* (IP12)

In contrast, work tasks related to problem-solving and creativity that require joint discussions were perceived as less suited for telework. This could for instance concern development of new services or products where colleagues need to get together and “brain storm” ideas.

*“If you are to bring forth something creative, you want to be able to interpose into each other’s speech, and use body language and perhaps show something with your hands and draw something on a whiteboard/…/It’s like, when the conversations become more of human interaction and less of only communicating about things.”* (IP6)

In this connection, especially meeting new colleagues “in real life” stood out as important in order to get to know each other, where nuances in expressions and body language as well as *“feeling”* each other were perceived more clearly and also created a more considerate affective tone, after which collaborating remotely worked out well depending on the type of work tasks.

*“Both emotionally and professionally, it’s very demanding in a zoom-meeting to “read” people as compared to if we had been in the same room. So, it’s been much more demanding to create a relation (with new employees/colleagues in telework).”* (IP13)

### Communication and social relations

A large part of the participants described that there is a risk of missing out on both formal and informal work-related information in telework. In this connection, they underscored the importance of ensuring that in particular formal information from the management that concern the entire workplace, reaches everybody in the staff when working from home.

*“You have to work hard to make sure everybody gets the same information.”* (IP1)

This experience was however not shared by all participants, where some instead found it easier to connect and share information during telework.

*“We had colleagues all over Sweden and you could just check-in for half an hour and you were supposed to do it every day. And it goes really fast and you can gather people from a large area.”* (IP2)

The risk of missing out on information, but also socializing with colleagues, was according to some of the participants, dealt with through active use of digital solutions, mainly in terms of scheduling regular meetings. The experiences of digital meeting formats however varied among the participants. Some expressed that communication and socializing during telework even increased as compared to before the pandemic, when at the workplace, and that it worked very well.

*“I think the relations actually have improved, just because we see each other more often on Teams than we did at the workplace.”* (IP15)

Others perceived that it did not feel natural or relaxed to meet digitally, and that especially the efforts to maintain some kind of socializing therefore was ended. This lack of communication and social relations in telework, digitally but also physically, was expressed to create a sense of loneliness and isolation.

*“To spontaneously talk about something work-related during a coffee break, that sort of things falls away (in telework)./…/We tried that (digital coffee breaks), but it never worked, it was so stiff.”* (IP7)

In this context, some participants expressed the role of managers in organizing and supporting meeting structures that enabled social connection and exchange, especially regarding sustaining the employee-manager relationship.

*“Our managers have re-scheduled their meetings with the staff as they want us (employees) to feel seen and grow even if they don’t bump in to us.”* (IP3)

For some participants, mandatory telework during the COVID-19 pandemic however rarely played a role regarding social relations. This could be explained by the fact that they were either freelance workers who did not have a specific workplace and/or colleagues or that their closest colleagues did not work at the same physical workplace. This led to that they were used to communicate, share information, as well as socialize digitally.

*“I don’t have any colleagues at the workplace, no one of those I work with is at the same office as me. So, being at the workplace doesn’t mean socializing in that way. I mean, if I am having a coffee break with my colleagues, I do that on the computer anyway.”* (IP5)

*“I have lost the social context to my company, physically. But digitally, I haven’t lost contact with those I work with a lot.”* (IP12)

Taken together, this theme illustrated that in telework, the working day was either filled with digital back-to-back meetings or alone time spent on tasks that require focus and concentration. This contributed to an overall sense of both high work intensity and loneliness in telework.

### Faces of flexibility: freedom and balancing boundaries

This theme highlighted experiences of increased freedom and self-determination in the organization of work, as well as strategies for navigating the boundaries between work and nonwork during mandatory telework. Two sub-themes were identified. The first sub-theme denoted the role of flexibility in telework for self-organization of one’s work. The second sub-theme revolved around issues concerning individual preferences as well as collective norms regarding the management of work-nonwork boundaries, and related perceptions of work-life balance.

### Time-spatial flexibility

Overall, the participants expressed an increased degree of freedom in daily life during mandatory telework due to a high level of time-spatial flexibility in the organization of one’s work. In particular, being able to decide for oneself when to perform work during the working day and week made it possible to organize work according to one’s preferences as well as adapt work to non-work commitments and vice versa.

*“I could work effectively until 2 o’clock when my daughter comes from school, and then I worked more the other weeks (as single with children at home every other week). And it’s great to have a job where that’s possible! Everybody can’t decide their work hours for themselves.”* (IP11)

Moreover, this increased sense of freedom was related to time saved, in particular in relation to not having to commute, that instead could be spent on other activities.

*“I have a long commute to work. 40 minute walk to and from the bus, and then one hour on the bus./…/ I don’t work an hour less, but as I don’t have to commute I’ve felt that there are more hours per week where I can do other things than working.”* (IP3)

However, the analysis also showed another side of flexibility with potential implications for what freedom in this context might entail. Specifically, flexibility enabled working longer hours, which therefore implied the “freedom” to work more. This, in turn, put pressure on the individual to not only being able, in terms of having the freedom, to decide when, but also having the ability to decide how much, to work.

*“The computer is always on, so then you can always do a little more and it easily gets more than eight hours a day, considerably more hours, and considerably more hours during weekends too./…/Sometimes there’s the risk that I end up on 12–14 hours in a day…”* (IP13)

For some participants, a high degree of flexibility during mandatory telework also led to a re-shaping of how one thinks about what constitutes work and thus, working time. Whereas before the pandemic there was the tendency to view working time strictly in terms of hours spent at the workplace or in front of the computer, this had changed during mandatory telework. Participants described how they had become *“more generous in how I think in terms of working time.”* This could mean that just thinking about work during nonwork time now was regarded as actual work.

*“I haven’t been sitting in front of the computer, not documenting, but I have used my brain to think about it when I was doing the dishes./…/So, now I’m thinking “I’m going to add that as 20 minutes of working time.”* (IP5)

### Work-life boundaries and balance

Regarding the management of work-nonwork boundaries during telework—although a few participants expressed a preference for integrating work and nonwork—a majority preferred to keep work and nonwork separated, that is, segmentation. Both boundary management preferences could be understood as means to get work done, although the perceptions of how this was best achieved varied. For instance, participants who preferred integration expressed that blending work and nonwork helped them in keeping up with work tasks, which in turn, enabled them to let go mentally of work during work free time, that is, psychological detachment, or switching off. Those with a preference for segmentation, in contrast, perceived that keeping strict boundaries between work and nonwork was important in order for them to know when they were done with work. For them, flexibility in telework was something they used mainly when something in their private life needed to be taken care of during standard work hours.

*“I like cleaning up (get work done) so it doesn’t keep lying on the desktop and it just gets worse. It feels better responding to something and then pass it forward to someone else, than have it laying around, with expectations, over the weekend and then deal with it on Monday.”* (IP14)

*“When I work, I want to work. Then, sometimes, I might have to do private errands, and then you just have to work longer than you had expected.”* (IP2)

A majority of the participants, especially those with a preference for segmentation, described various strategies used in order to successfully navigate their work-nonwork boundaries. These strategies could include having a separate workspace at home if possible, or having private versus work-related technological devices, which helped in keeping the spheres of work and nonwork separated.

*“I have work equipment and private equipment./…/So I can’t sit and watch a movie and then just switch back to work. It’s not possible, because it’s not the same gear and equipment./…/So, at five o’clock I turn off my work equipment.”* (IP16)

Another common strategy was to have daily routines similar to when working at the workplace, including good planning of both work and nonwork activities, which helped in having regularity and a clear structure of the working day and week. For some, these strategies even included symbolical actions, such as changing their clothes, that facilitated the transition between work and nonwork, thus underscoring when one ended and the other started.

*“You sort of have a routine not to sit in just your pyjamas in front of the computer, and then go to bed without “showing up” at work./…/Every morning I get up, have breakfast, take a shower, get dressed. When my working day is over, I can take off the clothes I wore during work in order to transport myself back home, home in sweatpants. Because it’s so important for me that work is work.”* (IP13)

A shared experience among the participants was that work-nonwork boundary management in telework could be problematic, as compared to when working at the workplace, in terms of keeping work from spilling over into nonwork, resulting in blurred work-nonwork boundaries, and the other way around.

*“When you’re to catch a bus, you know you have to stop working before going to the bus, so that’s good. Close down (the computer), leave meetings and such. So, it can be quite a good ending point. At home, I can always work little more.”* (IP3)

This kind of work-to-nonwork transitions were perceived as involuntary, irrespective of one’s boundary preferences. As such, even if having a preference for integration, this does not mean that one wants to work all the time, not having any boundaries at all between work and nonwork. It simply means that one wants to decide for oneself when to work and when not to, although one does not have the desire or need for structuring the working day and week in a traditional way in terms of working during standard work hours and at the workplace.

*“What’s negative is that I also work during a Sunday. I work a Saturday night, Thursday…/…/So, it’s fleeting together a bit.”* (IP11)

*“But it’s definitely a blurry line between being (work) free and working, so it’s (telework) is not always positive./…/It’s a trap and an opportunity.”* (IP6)

Although work and nonwork did tend to merge, many participants did however not experience this as too difficult to manage. Mainly, this could be understood in terms of that they themselves perceived to be in control over their work-nonwork boundaries, rather than these being determined by external factors such as work demands. This perception of high boundary control furthermore seemed to be the result of previous experience of telework, but also from the job itself. In this way, experience provided a sort of training for working from home where one eventually learns to set boundaries based on own choices, which gave a sense of control.

*“I don’t feel that I have to do a lot more just because I can./…/But I’ve had the possibility of working from home since the 90’s./…/I have built resilience over a long period of time./…/… you need that training mechanism when working from home.”* (IP14)

*“Sometimes I have to do things outside standard work hours/…/…but with my experience, I’ve been so long in the game, that I have the possibility to steer and see what needs to be done./…/But it’s still a choice I make.”* (IP16)

For others, with less previous experience of telework and/or who described themselves to be passionate about their work, managing one’s work-nonwork boundaries was more of a struggle. During telework, with its endless possibilities for working whenever and however much, there was a realization that this was not a sustainable situation and that one had to set boundaries for oneself, and as such, gain, or take, control, in order to achieve a sense of balance between work and nonwork. The latter was described as essential as primarily the nonwork domain, family and friends, according to the participants, give both energy for work and balance in life.

*“It felt like I never came away from work, but was just immersed in it…then I put up these boundaries “No, this is a job, it’s a job that I love and can spend endless hours on, but it’s still a job, and my friends and family is what actually gives me all those things that gives the strength to deal with daily life, especially when it’s tough at work.””* (IP13)

In view of the above, perceived boundary control can thus be interpreted as the result of well-functioning boundary management in terms of the creation and maintenance of one’s preferred work-nonwork boundaries based on autonomous choices, which in turn, is needed in order to achieve work-life balance.

In addition to these individual boundary management styles as well as the perception of being able oneself to control (the transitions between) one’s work-nonwork boundaries, the participants described that in order to create and maintain one’s preferred work-nonwork boundaries, it was important with shared, or collective, boundary norms both at home and at work, where family members as well as colleagues and managers served as border keepers. This was particularly pronounced by those with a preference for segmentation, where daily routines when working from home, for instance regarding when to start and finish work as well as breaks, was facilitated if other family members also worked from home, whereas this was more difficult when being home alone.

*“We tried to keep it between 8 and 5 o’clock. We had a coffee break, and said “Let’s move downstairs.”/…/And then we tried to talk about other things (than work), and after ten minutes “Let’s go back upstairs and work.”, and the same thing with lunch.”* (IP12)

*“It’s easy to forget about lunch without routines, maybe it gets delayed./…/So, I’ve thought about that a lot, that I should get better, that more structure would make it easier at home (during telework).”* (IP1)

In particular, having children living at home was perceived as something that forced one to stop working, which in turn, enabled balancing work and nonwork.

*“…the family situation with the children, they need my attention, food is to be prepared and driving to leisure activities, so you have to keep a time schedule.”* (IP7)

This pattern was further underscored by participants who were single, either with shared custody of children who lived with the participants every other week, or singles without children in the household.

*“I don’t know…private life, work…what is what?/…/As single with kids every other week…thanks to that I am busy with solving daily problems at home, it makes you disconnect from work.”* (IP9)

In addition to support from family members in managing work-nonwork boundaries, norms regarding availability on work-related issues outside standard work hours was perceived by many of the participants as vital. A majority of the participants expressed that at their workplace, there was respect and support for undisturbed work free time. This could take the form of spoken or unspoken mutual agreements between colleagues about not contacting each other, unless in some sort of emergency, outside standard work hours.

*“We were quite good and respected that in my work group, that is, people don’t call each other in the evenings and during weekends asking “How was it now with that?”. No, then you wait until Monday instead.”* (IP10)

Even more important for enabling well-functioning work-nonwork boundary management was the attitude toward availability outside standard work hours held by managers. Specifically, managers who expressed segmentation norms and also helped employees set clear work-nonwork boundaries was found to play a crucial role for experiencing a balance of boundaries.

*“And then I had my boss who was in close contact with me to make sure it (work) didn’t spread over too much (into nonwork), so she put firm boundaries, and I was only allowed to work over one night a week, if needed.”* (IP3)

This kind of managerial boundary support was however not perceived by all participants. For some, achieving balance between work and nonwork, mainly in terms of not working too much overtime, was something that had to be achieved by one’s own efforts, even when this might go against organizational norms and expectations.

*“My company don’t have anything (support) for you as a private person…it’s very conservative/…/I have to take the work-life balance I can.”* (IP4)

According to a vast majority of the participants, given having access to the boundary support described in the above, telework was perceived as key in facilitating work-life balance. The freedom that mostly followed from high time-spatial flexibility in telework, depending on the management of one’s work-nonwork boundaries, enabled one to organize overall life in a way that made the pieces of the *“jig-saw puzzle of life”* fit together.

*“For me, working from home is an enormous opportunity to be able to balance both, work with private life.”* (IP10)

*“And having that 100 percent flexibility at home…there’s no problem taking the dogs for a walk and such…so I find that balance…I don’t know, it’s great, it really is.”* (IP14)

Taken together, this theme pointed to that self-organization of work according to own preferences as well as commitments in work and nonwork, and also support of collective segmentation norms, especially when expressed by managers, played a pivotal role for perceived boundary control and the experience of work-life balance.

### Leadership challenges: bridging the gap between employee- and organizational needs

This theme highlighted participants’ perceptions of various challenges that managers face in the wake of the COVID-19 pandemic in order to maintain both employee job satisfaction and performance. Two sub-themes were identified. The first relates to employees’ as well as managers’ needs, respectively, with regard to employee autonomy and the role of leadership in telework, and the power dynamics and potential conflict between them. The second sub-theme denoted perceptions of how managers can balance individual employee preferences for working from home with clear and transparent performance measures in order to ensure organizational productivity demands.

### Trust vs. control

After the COVID-19 pandemic mandatory on-site meetings were re-introduced, which by many participants was perceived as going back to a work mode characterized by” *having meetings just to have meetings.”* In many instances, this return to the workplace, often times only to attend meetings, felt unnecessary, especially as the meetings were perceived to mainly concern things that had already been communicated to all employees through email. Participants expressed a sense of that this return to the workplace was more about managers’ own need of regaining control over employees, which served to reproduce traditional power structures and uphold organizational hierarchies. The flexibility and freedom gained during mandatory telework was therefore perceived as threatened. This caused upset and conflict with the management, especially in light of that the vast majority of the participants expressed that they had worked at least as hard, if not more, and performed better, during telework as compared to working at the workplace.

*“And then, when we came back, people didn’t buy in to that control in the same way, because then (after the pandemic) it became easier for her (the manager) to execute the same control again. And that’s why I think it was pretty healthy that it became quite a lot (of conflict) around that.”* (IP5)

In this connection, the participants described how the employer pushed for the implementation of formal agreements on when to be at the workplace and when to work from home. These agreements could be highly individual as well as very specific and elaborated, for instance being allowed to work from home either 2 days a week, or 4–8 days a month. In spite of this, many participants said that they had about the same freedom as before the pandemic when it came to working from home, but that it now had become more pronounced that one had to communicate to one’s manager when working from home. Yet other participants expressed that the employer had decided on agreements regarding workplace attendance and telework, but that the employees had neither signed nor adhered to them.

*“We continue to work remotely, most of us. We got an agreement, but I don’t think anyone signed it. It was 50 percent telework and 50 percent at the workplace after the pandemic. But we work more from home without having signed the agreement.”* (IP11)

Those participants who were managers perceived that norms reflecting a right to work from home had developed during the pandemic, which contributed to employee feelings of injustice when now being called back to the workplace. These opposite perspectives and needs held by employees on the one hand, craving autonomy and trust-based leadership, and managers on the other, who argued that they had the responsibility in whether employees could work from home or should be at the workplace created tension and power struggles.

*“When you give something it quite quickly becomes the norm./…/You need to have that in mind as a manager “What is it that I am implementing now?” If I, in the* future*, would say “Now we have to go back to the office” it’s would be perceived as a punishment./…/ But the manager must be able to decide “Now everybody is going to the office” and then you have to be there.”* (IP12)

In order to meet employees’ different needs for being at the workplace and/or working from home, and as such, not having to force people back to the workplace which might create conflict, a call was made by managers for technical solutions that enable hybrid work. In this way, it would be possible for all employees to participate on work-related issues as well as attend meetings independently of work location. The perceived lack of hybrid solutions could be understood as a question of organizational culture, and particularly managers’ norms and expectations regarding workplace attendance, but also as insufficient knowledge on how to implement and use technology.

*“I think it’s still a lot like “Can you hear me?”. No, technical problems and that there is no dialogue with those who are on zoom, whom we in the room can’t see without anybody asking a person something. We loose the natural conversation, and there I think our workplaces…we should…we could develop much more in order to make it work for everybody to work remotely.”* (IP 7)

Developing technical solutions for hybrid work as well as cultivating a telework work-friendly organizational culture was, in spite of the general call for employees to return to the workplace, regarded as crucial by managers. This was explained by the fact that the possibility of working from home was perceived as increasingly important, even more so than salary, in the recruitment of new employees. As such, both hybrid work and the possibility of working from home was seen as major factors for an organization’s attraction.

*“You can agree on a lower salary, or the same salary, as long as there is this (possibility of working from home). I think it’s a transition period. Then comes hybrid, then working from home is just a hygiene factor. In two years, it will be.”* (IP12)

### Employee preferences and performance measurement

From the perspective of employees, there was a common experience among the participants that effectivity and performance are independent of work location, and often times even higher in telework. Therefore, it was stated to be of higher relevance that the management consider, and adjust the work situation to, individual preferences rather than to just call all employees back to the workplace after the pandemic.

*“Just because you go to the workplace it doesn’t mean that you’re 100 percent effective.”* (IP8)

*“I think you have to adjust it individually to each person working from home.”* (IP11)

Participants holding a managerial role, in turn, expressed that telework entail freedom under responsibility which require that employees have the capacity to self-regulate their work effort in order to maintain a high job performance. In this connection, it was further expressed that it is not sufficient that employees themselves perceive that they perform equally well, or better, when working from home. Hence, in order to enjoy the freedom accompanying telework, employees also need to be aware of, and accept, that the management conduct individual follow-ups in terms of task specific performance measurement in order to ensure that organizational productivity demands are met.

*“You have to follow up to ensure that the productivity is the same and you have to see who is slowing down productivity. If someone doesn’t make it, but watches Netflix all day, I am not going to be able to prove that, I can only show what* tasks *this person actually does, so it has to be tasks that you somehow can condition and measure./…/And then you also has to accept, as an employee, that someone is checking up on you like that.”* (IP12)

Establishing structures and routines for this new way of working would however, according to the managerial participants, require a considerable effort. This would for instance include an increased level of specificity of work assignments and tasks in terms of what, and how much, to do, and in what time frame, as well as the employment of concurrent administrative report- and follow-up systems, which would enable performance measurement independent of work location. Managers would also have to pay specific attention to each employee working from home, conducting recurrent personal follow-ups targeted at employee perceptions of how work is going, what they are doing in their work, as well as general feelings regarding their work situation, in order to be able to support employees in creating the daily structure needed for working from home.

Taken together, this theme revolved around issues related to the power dynamic between employees’ call for freedom and trust-based leadership as to meet their preferences for working from home versus the exercise of organizational control as displayed by managerial demands of workplace attendance. Suggested solutions to reconcile the needs of both parties concerned the cultivation of an organizational culture embracing telework further development of technical administrative systems that enable individual and task specific performance measurement independent of work location.

### Survive or thrive? Telework and quality of life

This theme revolved around participants’ overall experiences of telework during the COVID-19 pandemic as related to quality of life. Two sub-themes were identified. The first concerned the role of telework for social relations and leisure time activities outside work. The second sub-theme focused on health-related experiences during telework.

### Leisure and lifestyle

Many of the participants expressed the importance of prioritizing social relations in personal life and leisure activities during telework, and that they had done so to a higher degree than before the pandemic. In particular, cultivating nonwork social relations was deemed crucial as the general experience among the participants was that one easily gets lonely and isolated when working from home. As such, enriched nonwork relations acted as a buffer against the loss of work-related social interaction. In addition, there was the shared experience that telework, due to increased time–space flexibility, and subsequent time saved, contributed to having more both time and energy for leisure activities.

*“We really had the need to meet and take that walk. It was very valuable to me, not feeling so lonely.”* (IP7)

*“I play a lot of golf and padel, and that’s where I get the social, personal contacts (during the pandemic). It becomes like I prioritize them much more than before. And I have much more energy for that.”* (IP1)

For co-habiting participants, especially those with families, nonwork social life was however experienced as more or less the same during telework during the pandemic. In this way, family life with its routines and daily chores, seemed to protect participants from the otherwise common feelings of loneliness and isolation accompanying telework.

*“I don’t think it (telework) has affected my social life that much. But now, we’re a family with small children. Life is quite messy where we sit together with the kids and have spaghetti days on end, it’s all pretty much the same.”* (IP6)

In contrast, participants with less opportunities for social interaction in personal life during telework, displayed feelings ranging from loneliness to depression.

*“During telework, in the evening or the weekend, I realized that it was a long time since I spoke to someone.”* (IP3)

*“It became an isolation that was very, very difficult.”* (IP5)

### Recovery and wellbeing

A general experience shared by a majority of the participants was that they perceived lower work-related stress, and subsequent improved recovery and wellbeing, during mandatory telework. This was not due to that they worked less, but rather, more focused and effectively, but also, due to the increased time–space flexibility gained in telework, to having more time for relaxation and recovery when not having to commute to work.

*“For me, it’s a big difference. I feel much more calm, less stressed.”* (IP1)

Experiences of improved sleep were also common. As participants got longer sleep in general, irrespective of whether going to bed earlier or staying up later, this also benefited recovery and health.

*“We made earlier nights, and I sleep longer, and that’s something we are trying to keep now. We feel that it was good for us.”* (IP3)

*“I stay up longer in the evenings. I do not have to get up early, 30 min before starting work is enough, and then I just have a cup of coffee.”* (IP4).

On a more negative note, many participants described challenges related to keeping up with physical activity and also that their eating habits had become poorer during telework, resulting in a sedentary lifestyle and weight gain.

*“In theory, I could go to the gym more often, but usually that doesn’t happen as I have too much else to do. What happens instead is that, if I don’t go to work, the risk is that it becomes more sedentary./…/It could be that, some days, I haven’t even gone out through the door, and that’s not good.”* (IP6)

However, some participants expressed that their physical activity rather had increased, and that eating habits had improved, as one could exercise whenever as well as make healthier lunch at home than when at the workplace.

*“It has become much, much more (exercise). Better food, and significantly improved fitness.”* (IP4)

More often it was however not the case that participants’ lifestyle habits had either improved or got worse, but rather that it was a mix of both. For instance, engaging in increased physical activity could not make up for more snacking between meals when working from home, which resulted in weight gain.

*“I was actually very meticulous about getting some exercise, but it didn’t compensate for the proximity to the fridge.”* (IP9)

Regarding sickness absence, there was according to the participants a decrease during mandatory telework. This does however not necessarily entail that people were less ill, but rather that it is easier to work although one is ill when working from home. In this connection, it was perceived as difficult to decide when one is ill “enough” as not to work during telework, but also that one’s work tasks does not go away when on sick leave but rather piles up. Thus, many participants, if not too ill, still worked in order for their work load as not to become unmanageable when returning to work after sick leave.

*“I sometimes work at occasions when I perhaps should have called in sick. So I find that quite difficult, to feel when I am ill enough, not even having the energy or strength to turn on the computer or attend these meetings.”* (IP5)

*“If I am ill, I usually log in at the computer from home and do what needs to be done because I know that it’s still there when I get back.”* (IP11)

Furthermore, although many participants stated that they themselves rarely are ill, they expressed that it was none the less common to work when ill in general during telework.

*“You can be in a Teams meeting and half of all have a fever of 38 degrees./…/If you don’t have pneumonia or stomach flu or something, you often sit in remote meetings with a bit of a cold in the body, and you notice that many of the others do too.”* (IP6)

This attitude of working although being ill, was however not shared by all participants. There were also those who expressed that they, irrespective of working from home or not, call in sick to work if ill. This choice depended both on the perception that sick leave was considered to be vital for recovery, but also that it was perceived as important to maintain a high level of performance in work, which might not be the case when working while being ill.

*“I think that if you are ill, you are ill, and it’s (sick-leave) about getting the recovery.”* (IP13)

*“If I have a headache I don’t work, then I sleep instead. I will not just “sit time off”, I want to perform when I work.”* (IP14)

Another explanation for the lower sickness absence during telework put forth by the participants was that actual illness was indeed rarer due to that colleagues did not infect each other as before the pandemic, when it was also fairly common to go the office although ill. Hence, sickness presence tended to be high even before the pandemic, with the difference being that during telework people were not infecting each other.

*“If someone is ill, and they otherwise would have come to work but now stays at home, then the rest of us don’t catch it.”* (IP1)

Taken together, this theme highlighted the importance of variation in daily life, cultivation of nonwork social relations and engaging in leisure activities, preferably physical and/or outdoor activities, for overall wellbeing and health in telework. Furthermore, sickness absence seemed to decrease in telework, but often times at the expense of increased sickness presence.

## Discussion

This study explored employees’ experiences of telework, to varying degrees, during and after the COVID-19 pandemic in Sweden, aiming to understand the role of telework for overall job satisfaction, wellbeing, health, and WLB. Additionally, it examines how employees navigate challenges and embrace possibilities that they encounter in both the work- and nonwork domains. Such knowledge is important with regard to the future working life but also in order to enhance social resilience (Alizadeh et al., 2023). The fact that the current study was conducted in a country where no lockdowns were applied and most of the participants continued with work arrangements that combined telework and on-site work after the pandemic allows enhanced understanding of pitfalls and opportunities of telework combined with on-site work which has become globally popular after the pandemic.

In the present study, five overarching themes were identified illustrating both challenges and opportunities in the work- and nonwork domains related to full or partial telework. The first theme concerned the importance of having ergonomic- and technological work supplies as well as a separate workspace in the home. The second theme related to increased work efficacy but also isolation due to reduced socializing with colleagues. The third theme revealed the role of increased work flexibility and perceived boundary control for WLB. The fourth theme showed that employees would like to continue to telework, in particular when combined with a few-days on-site work, which highlighted that managers need to strike a balance between trust-based leadership and performance measurement. Finally, the fifth theme highlighted individual variations in quality of life between workers who telework in varying degrees based due to personal and work factors. It was also found that sickness presenteism increased because of telework.

Overall, the participants expressed that telework provided benefits in terms of more flexibility and greater freedom to plan and perform work, which in turn entailed more freedom and time for leisure activities and socializing with family and friends. This, in turn, was perceived as highly positive for health, for instance in terms of less stress and improved sleep, as well as for wellbeing, and WLB and also increased work efficacy, which is in agreement with earlier findings (D’Abundo et al., 2023; Lindgren et al., 2023; Šmite et al., 2023). The latter was explained by improved focus on one’s work tasks due to absence of office interruptions and socially imposed breaks as well as not having to commute - results that that also previously have been described as benefits of telework (Espersson et al., 2023; Russo et al., 2023; Šmite et al., 2023). In contrast to previous work, which has suggested that combining telework (during the COVID-19 pandemic) with care of children resulted in increased WFC (Craig and Churchill, 2021; Miller and Riley, 2022; Zhang et al., 2020), this was not the case in our study. One possible explanation for this is that preschool and nursery care as well as to a large part the schools in Sweden were kept open during the pandemic. Although this qualitative study did not enable aggregation of experiences by demographic factors like age, gender, and family type, the findings indicated that these factors seem to play a role for the overall experience of quality of life while working from home, which has also been pointed out in previous studies (Awada et al., 2021; D’Abundo et al., 2023).

On a more negative note, telework, independent on its degree, often implied working longer hours and work intensification as well as increased boredom and loneliness during work hours. The latter was due to lack of spontaneous work-related interactions, which instead were limited to pre-scheduled digital meetings as well as restricted to a limited and rather selective number of colleagues. Physical health issues related to lifestyle in terms of poor eating habits and weight gain as well as increased sickness presence were moreover common. Also, musculoskeletal problems related to poor ergonomics due to lack of appropriate office equipment in the home were prominent. Further, the lack of a separate workspace in the home was considered a hindrance for creating and maintaining clear boundaries between work and nonwork, which in turn, was important for WLB. These findings are in line with previous studies that highlighted the risks of telework for health and WLB (D’Abundo et al., 2023; Lal et al., 2023; Šmite et al., 2023), as well as the importance of having a workspace at home that is properly prepared for carrying out professional tasks (Santos and Pereira, 2023).

The findings showed a high degree of individual variations with regard to both the extent and quality of social relationships with colleagues. Some participants reported that work-related social interactions had become more scarce compared to before the pandemic when mainly working full-time from home. For many participants, this lack of social relationships at work had implications for feelings of loneliness and poor wellbeing, whereas others expressed increased exchange and improved relationship quality with colleagues. The latter is in agreement with prepandemic studies showing that voluntary teleworking affect workplace relationships between colleagues positively (van der Lippe and Lippényi, 2020). Our findings in this context corroborate that in particular managers played a vital role for initiating and cultivating social exchange- and relationships in telework where employees work from home in varying degrees. On a related note, managers were also found to serve as so-called border keepers (Clark, 2000) having an important support function, with regards to work-nonwork boundary management. For instance, participants who perceived managerial support in setting clear boundaries between work and nonwork expressed that this prevented work from spilling over into personal life, which resulted in working less overtime and was also beneficial for WLB, recovery, and health. In contrast, participants who perceived high expectations to be available on work-related issues outside standard work hours expressed more blurring of work-nonwork boundaries, with negative implications for WLB, and health, which has also been shown in earlier studies (Brough et al., 2021; Chan et al., 2022; Miller and Riley, 2022). Further, previous research has shown that telework is more easily implemented by organizations characterized by a low need for control (Hamilton, 2002; Harrington and Santiago, 2015; Standen, 2000) and that there are positive associations between a non-hierarchical organizational culture and employee health, wellbeing, job quality, and happiness (Ficarra et al., 2021; Junça Silva and Coelho, 2022; Kwon and Jeon, 2020). It has also been found that the risk for low job satisfaction increases when there is lack of congruity between employees’ desired- and actual organizational culture (Mesfin et al., 2020). These earlier findings are important in the context of the current study as many of the participants expressed dissatisfaction with being called back to the workplace after the COVID-19 pandemic, where the reason for this was mainly due to the re-installement of mandatory on-site meetings by the management. This was perceived as reflecting managers’ own lack of trust and need of regaining control over employees, which was interpreted as serving to reproduce traditional power structures and uphold organizational hierarchies. Given the latter, the participants expressed that it is important for managers to enact trust-based leadership, rather than control, as well as to be sensitive to individual employees’ telework preferences and work-nonwork boundaries in general. Finally, the increased sickness presence, and related perceptions of health when teleworking fully or partly, that we found in this study is supported by previous studies showing that the COVID-19 pandemic affected perceptions of health at work (Kawada, 2021; Ruhle and Schmoll, 2021; Steidelmüller et al., 2020).

Overall, the findings in our study highlight that the Swedish strategy, which has been described as implying “mild law and high individual responsibility” and freedom of decision with more lenient public health restrictions allowing telework arrangements to varying degress during the COVID-19 pandemic, played a mainly positive role for employees’ experiences of their overall job satisfaction and job characteristics, wellbeing, health, and WLB (Ludvigsson, 2020; Pashakhanlou, 2022). Indeed, recent results by Benke et al. (2020) provide further support to that although negative experiences in terms of for instance feelings of increased loneliness and isolation were expressed by the participants in the present study, the many concurrent positive experiences could be interpreted as resulting from the lower level of restrictions practiced in Sweden.

### Strengths and limitations

The present study includes 16 participants, with data collection concluding upon reaching thematic saturation. As such, the sample can be considered being sufficient for an in-depth qualitative interview study. It is also consistent with recommendations for in-depth qualitative interview studies and provides a robust basis for exploratory analysis. The sample of participants, drawn from a large longitudinal study approximately representative of the Swedish working population, further strengthens the credibility of the findings. It provides diversity regarding age, gender, family type, living area, industry, and degree of telework during and after the pandemic. Also, the current study, as being conducted in a country with no total lockdowns and generally more lenient public health restrictions during the COVID-19 pandemic, allows to explore the role of telework in a different context than most previous pandemic research. Furthermore, the study extends knowledge on work arrangements that combine telework with on-site work, given that the degree of telework varied between participants both during and after the pandemic.

The interviews lasted between 40–60 min and open-ended questions were used in order to obtain answers that are rich and can be further elaborated. Another aspect to consider is that the interviews were performed via Zoom or telephone and not through face-to-face contact. This might have both benefits and limitations (Archibald et al., 2019). For instance, in interviews online, participants may be more responsive than face-to-face participants (Salmons, 2016), but this format can also create a larger communication distance and the interviewer may not capture gestures and facial expressions as in face-to-face interviews. Another shortcoming might be that the interviews were conducted approximately 1 year after the pandemic recommendations for telework were lifted. Thus, a certain recall bias cannot be excluded. Participants might remember telework during the pandemic as both more positive and negative, respectively, than they experienced it at the time being or mix up current feelings and experiences with those during the pandemic. Still, they could well report their experiences of telework after the pandemic. Finally, it is also important to note that this is a qualitative and exploratory study and, as such, the establishment of causal links is not possible. Future research, could quantify the relationships using larger samples.

### Practical implications and recommendations

The results of the present study provide useful information that can be extrapolated to work arrangements that combine telework with on-site work in the post-pandemic era, and as such, help guide organizations in the successful implementation of such work arrangements and also determine appropriate actions to improve overall job satisfaction, wellbeing, health, and WLB for employees that combine telework with on-site work.

Although its many benefits, telework imply challenges for employees as well as to organizational sustainability and human resource management. For managers, the latter can be difficult if they feel reduced possibilities for supervising employees who are working fully or partly from home. In that case, there is a risk that an active remote leadership can be perceived as controlling and hierarchical by the employees, with potentially detrimental implications for job satisfaction, work engagement, and even turn-over. There is also a risk that employees who are on-site receive more attention from managers and that teleworkers are “punished” through poorer access to information and work tasks as well as receive poorer evaluations on performance, which worsens pay and career paths.

In order for future telework to be sustainable, organizations would benefit from providing employees with equal pre-requisites in terms of home-based ergonomic- and techonological supplies, and in particular, implementing leadership based on trust, enabling work-related social relations and connection. Additionally, it is important to reduce feelings of social isolation, as well as support the setting of clear work-nonwork boundaries, aided by the development of organizational segmentation norms, e.g., introducing clear policies on when employees are required to answering work-related demands. A shared understanding by employees and employers is also essential to address the evolving dynamics in telework ensuring that all employees are treated fairly, and have equal career opportunities. Managers and HR would need to accommodate changes in organizational strategies as well as in HR policies to provide effective organizational support and prevent risks for employees’ health, wellbeing and WLB. For instance, implementing supportive leadership practices that ensure cohesion, regardless of whether employees work fully or partly from home, by fostering opportunities for direct communication, collaboration and social support, can help maintain both the quantity and quality of social interactions. In this connection, it must be taken into account that the opportunities and challenges of telework in terms of both prevention and leadership practices differ from those of full-time telework and full-time on-site work. Managers must therefore consider the contextual factors of the respective work situation when supervising teams that work as well in home-based as in on-site offices, e.g., by acknowledging that not all employees may be available at the same time due to different work environments. Also, manager should try to respect individual employees’ preferences for on-site, full or partial telework, work-nonwork boundaries, and hence adapt their communication, leadership practices and strategies accordingly to each context.

## Conclusions and suggestions for future research

This qualitative study shed light on the role of telework on employees’ work life and WLF during and after the COVID-19 pandemic in Sweden. Overall, increased flexibility in work, combined with well-functioning work-nonwork boundary management and related supervisor support, entailed more time for leisure activities and nonwork social relationships, which was perceived as positive for wellbeing and WLB. Although work efficacy was high in telework it also increased work intensification, work-related isolation, ergonomic problems, and sickness presence. Future research would benefit from quantitative, longitudinal studies assessing the impact of telework over time on working conditions, occupational safety and health, and related gender- and social inequalities in health.

## Data Availability

The datasets presented in this article are not readily available because the data are confidential. Requests to access the datasets should be directed to constanze.leineweber@su.se.

## Appendix

**Table A1:** Participant characteristics.

IP	Gender	Age	Family type	Region	Industry	Telework after the COVID-19 pandemic
1	Female	57	Divorced/No children living at home	North Sweden	IT/University competence	>75%
2	Male	49	Married/Children living at home	Central Sweden	Engineering	>75%
3	Female	64	Married/No children living at home	North Sweden	IT/University competence	25-75%
4	Male	59	Married/No children living at home	South Sweden	Sales	>75%
5	Female	35	Married/Children living at home	Central Sweden	Engineering	<25%
6	Male	49	Co-habiting/Children living at home	Central Sweden	Culture	>75%
7	Female	44	Single/Children living at home	North Sweden	Teaching	>75%
8	Female	60	Divorced/Children living at home	Central Sweden	Engineering	<25%
9	Male	52	Divorced/Children living at home	Central Sweden	Teaching	25-75%
10	Male	48	Married/Children living at home	North Sweden	Engineering	<25%
11	Female	43	Single/Children living at home	North Sweden	Social work	25-75%
12	Female	55	Married/Children living at home	Central Sweden	Manager in finance/sales/marketing/personnel	>75%
13	Female	42	Married/Children living at home	South Sweden	Office clerks	25-75%
14	Male	52	Married/Children living at home	Central Sweden	IT/University competence	>75%
15	Male	44	Married/Children living at home	Central Sweden	IT/University competence	>75%
16	Male	57	Single/No children living at home	Central Sweden	IT/Sound/Lightning technology	<25%
